# The Role of Associative Cortices and Hippocampus during Movement Perturbations

**DOI:** 10.3389/fncir.2017.00026

**Published:** 2017-04-19

**Authors:** Matthew S. D. Kerr, Pierre Sacré, Kevin Kahn, Hyun-Joo Park, Mathew Johnson, James Lee, Susan Thompson, Juan Bulacio, Jaes Jones, Jorge González-Martínez, Catherine Liégeois-Chauvel, Sridevi V. Sarma, John T. Gale

**Affiliations:** ^1^Department of Biomedical Engineering, Johns Hopkins UniversityBaltimore, MD, USA; ^2^Center for Neurological Restoration, Cleveland ClinicCleveland, OH, USA; ^3^Department of Neuroscience, Cleveland ClinicCleveland, OH, USA; ^4^Epilepsy Center, Cleveland ClinicCleveland, OH, USA; ^5^Institut National de la Santé et de la Recherche Médicale UMR 1106, INSMarseille, France; ^6^Aix Marseille UniversityMarseille, France

**Keywords:** neuroengineering, motor control, robust motor control, SEEG, association cortices, hippocampus, P300

## Abstract

Although motor control has been extensively studied, most research involving neural recordings has focused on primary motor cortex, pre-motor cortex, supplementary motor area, and cerebellum. These regions are involved during normal movements, however, associative cortices and hippocampus are also likely involved during perturbed movements as one must detect the unexpected disturbance, inhibit the previous motor plan, and create a new plan to compensate. Minimal data is available on these brain regions during such “robust” movements. Here, epileptic patients implanted with intracerebral electrodes performed reaching movements while experiencing occasional unexpected force perturbations allowing study of the fronto-parietal, limbic and hippocampal network at unprecedented high spatial, and temporal scales. Areas including orbitofrontal cortex (OFC) and hippocampus showed increased activation during perturbed trials. These results, coupled with a visual novelty control task, suggest the hippocampal MTL-P300 novelty response is modality independent, and that the OFC is involved in modifying motor plans during robust movement.

## Introduction

The understanding of motor control has an extensive history within neuroscience research (Penfield and Boldrey, 1937; Matsuzaka et al., 1992; Schieber and Hibbard, 1993; Graziano et al., 2002; Popa et al., 2015). Most such research that includes neural recordings has focused on the primary motor cortex, pre-motor cortex, supplementary motor area, and cerebellum. However, the roles of the associative cortices and hippocampus in motor control and learning are poorly understood, particularly in the case of disrupted or perturbed movement. Ensuring movements are robust (successfully completed despite perturbations) poses a special challenge for the motor control system.

When a force perturbation is unexpectedly introduced during movement, the task becomes much more complicated (Cluff et al., 2014; Pruszynski, 2014). The sensory feedback stemming from the perturbation must be recognized as an aberration from anticipated input, engaging a novelty detection mechanism. A shift in spatial attention must occur, and in the case of large perturbations, the prior, now out-of-date, direct motor plan must be suppressed and a new plan created and executed. In addition, the motor system must identify and retain information related to the features of the external or internal aberration to adapt future motor plans (Bastian, 2008). Hence, we hypothesize the existence of event related potentials (ERPs) in the fronto-parietal pathway and hippocampus.

The fronto-parietal pathway is an internally differentiated but unified system and has been implicated in the use of sensory information to plan and execute goal directed tasks, particularly in adaptive behavior (Cole et al., 2014). Spatial information from the senses converges within the parietal cortex and is fed forward to the premotor cortex where it is integrated with information from the prefrontal cortex about action goals (Cole et al., 2014). The precuneus and intraparietal sulcus (IPS) are known to activate in conjunction with shifts in spatial attention, primarily studied in purely visual paradigms (Corbetta et al., 1998). The parietal regions have previously been shown to exhibit a scalp P300 response to visual stimuli requiring top-down attention (Bledowski et al., 2004). The orbitofrontal cortex (OFC) has been shown to play a key role, among others, in facilitating suppression of behavioral patterns that are no longer relevant (Rolls, 2004).

Additionally, the hippocampus plays a key part in novelty detection, possibly by comparing incoming stimuli to memories of similar situations (Kumaran and Maguire, 2006); its role as a generator of a P300 signal (specifically the MTL-P300) in response to novel visual and auditory stimuli is well documented (Solani and Knight, 2000). Moreover, the role of the hippocampus has been extensively studied with regard to memory, and is well positioned, both anatomically and physiologically, to be involved in encoding relevant associations between new stimuli and motor responses (Suzuki, 2007).

We hypothesize that the hippocampal novelty detection mechanism underlying the MTL-P300 response is modality independent and its activity will modulate with unexpected motor stimuli (i.e., force perturbations). In addition, we hypothesize the OFC is preferentially active when dealing with motor perturbations, reflecting modification in the motor plan, making it an important target for future studies of robust human movement.

To test our hypotheses, we leverage the wide anatomical sampling associated with stereo-electroencephalography (SEEG) to probe seven regions potentially involved in processing unexpected or “novel” motor perturbations. These areas were chosen balancing three criteria: membership in the fronto-parietal pathway (Cole et al., 2014), extensive innervation with hippocampus (Bird and Burgess, 2008)—shown to be involved in processing novel stimuli, and the frequency with which the regions are probed in the clinical environment (González-Martínez et al., 2014). All analysis was done based on careful anatomical parsing of clinically relevant implantation sites. For ethical reasons, researchers did not play any role in driving implantation patterns.

Specifically, we examine intracranial electrophysiological SEEG recordings from the fronto-parietal and hippocampal network during movements that were perturbed with unexpected forces. Twelve subjects performed one or both of two tasks: the motor task and a visual novelty control task. In the motor task, simple center-out reaches were made to four possible targets with 20% of trials experiencing a force perturbation. The visual novelty control task repeatedly presents the same common visual stimulus with 20% of trials showing a rare stimulus (the visual “perturbation”). Additional details of the experimental set-up are in the Materials and Methods section.

Coupling SEEG recordings with these experiments provides high spatial and temporal resolution neural data providing new insight into the function of the hippocampus and OFC during movement in humans, areas that are not traditionally studied as part of the motor circuitry. Both ERPs and high frequency activity (HFA) were examined in each structure mentioned above (Lachaux et al., 2012). ERPs capture the local field potential activity, which represents the average dendrosomic activity of presynaptic signals for large neuronal populations (Mitzdorf, 1985; Logothetis, 2003). Gamma responses (captured in HFA) represent multi-unit activity relayed by the interneurons on the pyramidal cells. As is frequently the case, HFA results show similar trends to the ERP data and for this reason are presented in Supplementary Materials.

## Materials and methods

### Participants

SEEG recording was performed in medically refractory epileptic patients in order to define the epileptogenic zone for possible resection (Talairach and Bancaud, 1973). The choice of electrode location was based on pre-implantation video-EEG recordings and was made independently of the present study. This study did not add any invasive procedure to depth SEEG recordings. Criteria for patients undergoing SEEG implantation were reviewed by clinicians to determine patient eligibility for enrolment in the current study. If the patient met study criteria, research staff not involved in the surgery implantation or post-surgical care contacted the patient for potential participation in the study. If the patient expressed interest in participating, the research staff would verbally review the written, IRB approved consent form. If agreed upon, the patient would sign the written consent and be enrolled in the study. A copy of the written consent would also be given to patient to keep. Experimental protocols were approved by the Cleveland Clinic Institutional Review Board. Methods were carried out in accordance with approved guidelines. Criteria required individuals over the age of 18 with the ability to provide informed consent and perform the behavioral tasks. Besides the behavioral experiments, no alterations were made to the course of clinical care. Patient details are listed in Table 1.

**Table 1:** **Study subjects' characteristics**.

**Patient**	**Sex**	**Age**	**Handed**	**Duration of epilepsy (years)**	**Epileptogenic zone**	**Tasks completed—Motor/Visual**
Subject 1	F	29	L	23	Left Insula	Motor (8–15 N)
Subject 2	F	60	R	8	Left Temporal	Motor (2.5–5 N)
Subject 3	F	37	L	12	Left Temporal	Motor (2.5–5 N)
Subject 4	F	36	R	36	Right Parietal	Motor (2.5–5 N)
Subject 5	F	32	R	13	Left Parietal	Motor (8–15 N)
Subject 6	M	24	R	3	Right Temporal	Motor (2.5–15 N)
Subject 7	F	34	R	5	Left Temporal	Motor (2.5–15 N) and Visual
Subject 8	M	23	L	17	Left Parietal	Motor (8–15 N) and Visual
Subject 9	F	53	R	18	Left Temporal	Visual
Subject 10	F	21	R	19	Right Parietal Occipital	Visual
Subject 11	F	48	R	6	Left Temporal	Visual
Subject 12	M	24	R	14	Right Temporal	Visual

### Stereoelectroencephalographic (SEEG) implantations

For each subject, approximately 8–13 stereotactically placed depth electrodes were implanted. The electrode contacts were 0.8 mm in diameter, 2 mm in length, and spaced 1.5 mm apart. Depth electrodes were inserted in either orthogonal or oblique orientations using a robotic surgical implantation platform (ROSA, Medtech Surgical Inc., USA) allowing intracranial recording from lateral, intermediate and/or deep cortical and subcortical structures in a three-dimensional arrangement (González-Martínez et al., 2016). The day prior to surgery, volumetric pre-operative MRIs (T1, contrasted with Multihance®–0.1 mmol/Kg) were obtained and used to pre-operatively plan electrodes trajectories. All trajectories were evaluated for safety; any trajectory that appeared to compromise vascular structures was adjusted appropriately without affecting the sampling from areas of interest.

### Electrophysiological recordings

SEEG electrophysiological data was acquired using a conventional clinical electrophysiology acquiring system (Nihon Kohden 1200, Nihon Kohden America, USA) at a sampling rate of either 2 KHz or 1 KHz for the motor task and 1 KHz for the visual oddball task. Behavioral event data were simultaneously acquired during behavioral experiments along with the SEEG electrophysiology and stored for subsequent analysis. All signals were referenced to contact affixed to skull. Archived electrophysiological data was not filtered prior to offline analysis.

**Figure 1:**
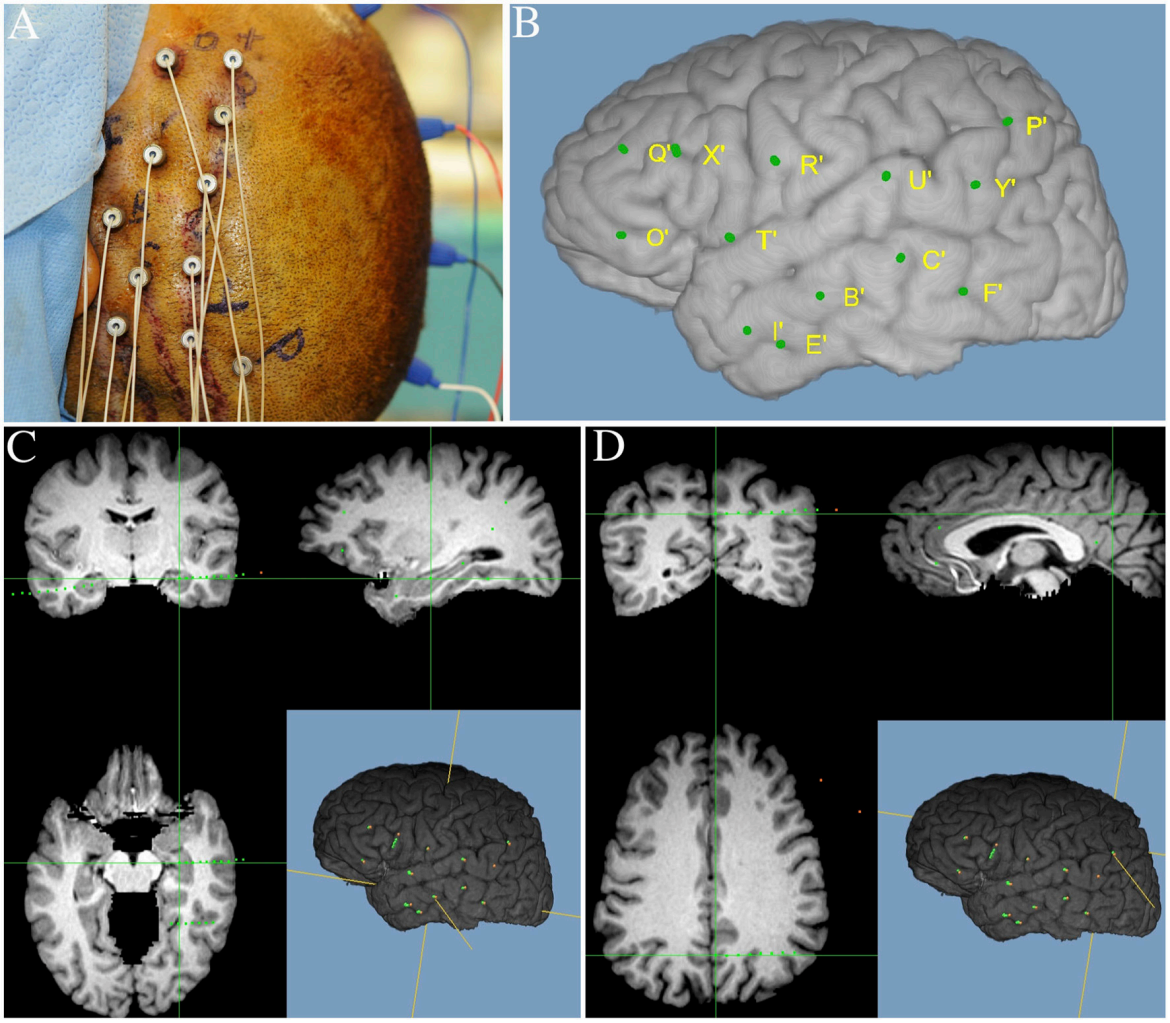
**Illustration of image merging procedure used to detail the anatomical location of each electrode contact. (A)** Final intraoperative aspect of left frontal-temporal-parietal SEEG implantation. **(B)** Three-dimensional MRI reconstruction showing details of superficial cortical anatomy and the relative position of the implanted electrodes. **(C)** MR images fused with postoperative SEEG implantation CT scan showing an example electrode targeting the left hippocampus. **(D)** MR images fused with postoperative SEEG implantation CT scan showing an example electrode, targeting the left precuneus.

Each patient had electrode contacts characterized according to anatomical location. The anatomical locations of all contacts were identified through inspection of post-operative imaging, requiring agreement by two clinical experts. An example of post-operative imaging contributing toward determining contact location is shown in Figure 1. Coronal and sagittal views were available for every contact. A list of implanted relevant regions is contained in Table 2. None of the recording electrodes selected for this study demonstrated epileptic activity (ictal or interictal) during the recording session.

**Table 2:** **List of implanted brain regions by task**.

**Anatomical region**	**Only Motor**	**Only Odd-Ball**	**Motor and Odd-Ball**
Anterior hippocampus	5	4	1
Posterior hippocampus	4	0	1
Orbitofrontal cortex	3	1	1
Anterior cingulate	2	1	2
Precuneus	6	2	1
Intraparietal cortex	6	1	1
Insular cortex	3	2	2

### Behavioral task

**Figure 2:**
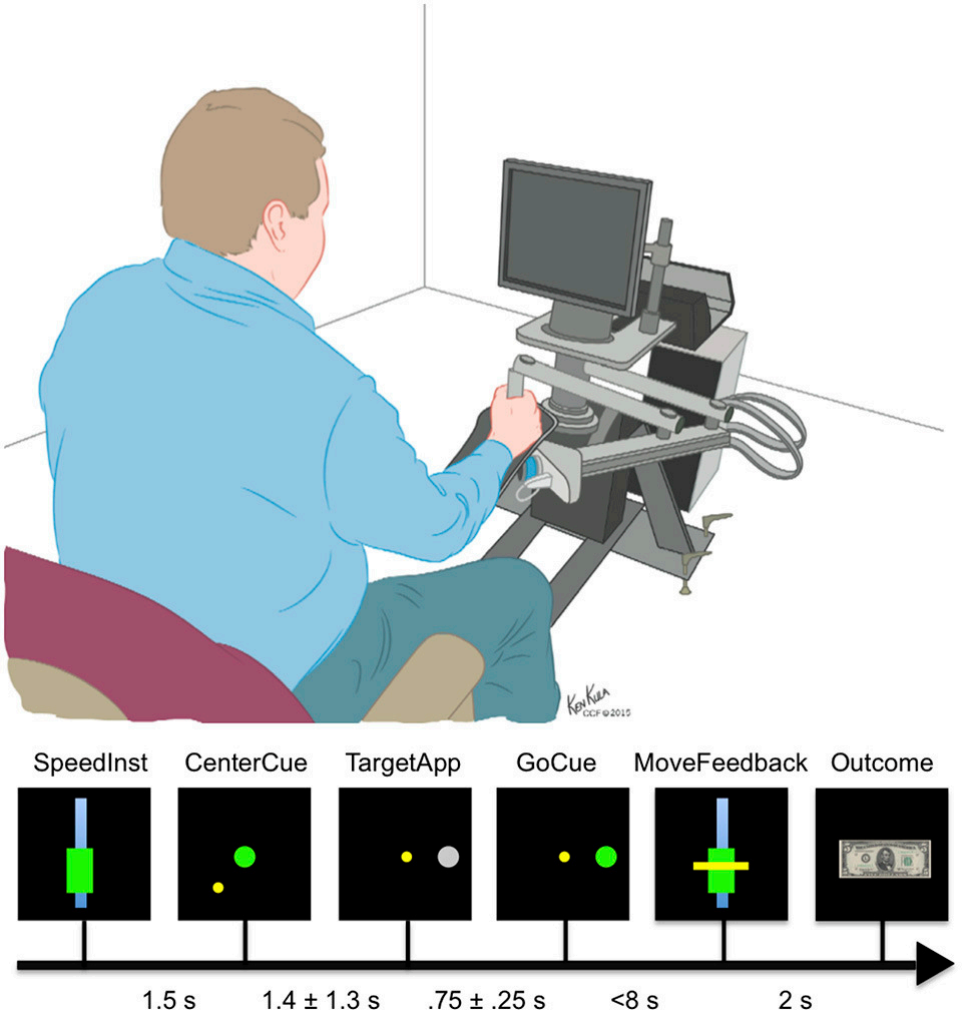
**Epochs of the motor task**. The subjects were given as long as necessary to center the cursor after the CenterCue. Times range between CenterCue and TargetApp represents mean and standard deviation of behavioral data.

Subjects performed the behavioral task in their Epilepsy Monitoring Unit (EMU) room while seated in a chair that was placed in front of the behavioral system (Figure 2), using methods previously described (Johnson et al., 2014). The behavioral system consisted of a computer presentation screen, an InMotion2 robotic manipulandum (Interactive Motion Technologies, USA), and a behavioral control system. The computer screen was used to present task stimuli to the subject and was located ~2 feet from the subject's sitting position. The robotic manipulandum is an FDA-approved device for motor recovery and allows for the precise tracking of arm position in a horizontal two-dimensional plane relative to the subject. This manipulandum allows the subject to control the position of a cursor during the behavioral task and was used to apply force perturbations to the subject during precise elements of the behavioral task. The behavioral control system consisted of a Windows-based laptop computer running MonkeyLogic (Assad and Eskandar, 2008) through a MATLAB® interface (MathWorks, USA).

### Primary motor task

The primary motor task that we administered was designed to conduct a study that investigates (i) movements at different speeds, and (ii) movements during unexpected force perturbations of varying amplitudes. In this manuscript, we present our findings on the behavioral and neural responses to force perturbations as we found that they are invariant to movement speed.

Prior to the start of the motor task, a calibration session was performed to scale the movement speed instruction cue presented during the main task. Details of calibration are in Supplementary Materials. The probability of all movement speed cues was equal across subjects and not the focus of the comparisons done here. For this analysis, the important feature is whether the movement is completed in a reasonable time frame, not whether it fell into very narrow bounds. Almost all movements made by subjects (See Supplementary Table 1) met that criterion.

The primary motor task (Figure 2) is based on a simple center-out reach task with four possible targets. On trials when perturbations were applied, a constant force was applied from the moment the movement started till the target was reached and held. The motor task consisted of seven epochs: speed instruction (SpeedInst)—visual cue that defines the desired range of speed to target, centering cue (CenterCue)—cue to move cursor to middle of screen, target presented (TargetApp)—first appearance of the gray target circle, go cue (GoCue)—target turns green, movement start (MoveStart)—onset of movement, movement time feedback (MoveFeedback)—presentation of the visual cue indicating the subject's duration of movement, and reward/failure (Outcome) cue—image presentation of the result based on matched or unmatched movement speed. MoveStart was also the point at which ~20% of trials had a perturbation applied.

The SpeedInst cue instructed either a fast movement or slow movement, and the targets appeared in any of four potential screen positions (up, down, left, or right relative to the screen's center). The subjects were allowed at most 8 s to acquire the target, after which the movement would be deemed incomplete, and the experiment would progress to the next trial. Before the 8-s limit, subjects could complete movements even if they fell outside of the instructed movement speed. Actual movement speeds were accepted to be a match if they fell within approximately 13 percentage points of the instructed speed. An American $5 bill served as the matching speed stimulus whereas the same stimulus with a red X over it served as the mismatched stimulus. If the target was not reached within the allotted time limit or if the subject began movement before the go-cue, the trial was marked as failed.

In trials with an applied force perturbation, the direction and magnitude of the perturbation was randomly selected from a uniform distribution where the range of force magnitudes varied within subjects but overlapped across subjects from a minimum of 2.5 N to a maximum of 15 N. We chose to alter the range of forces between subjects to mitigate the potential for overwhelming forces to any individual participant. All subjects were allowed to train on the behavioral task prior to experimental sessions until comfortable with rules, manipulandum, and force perturbations to be experienced.

### Visual oddball control task

**Figure 3:**
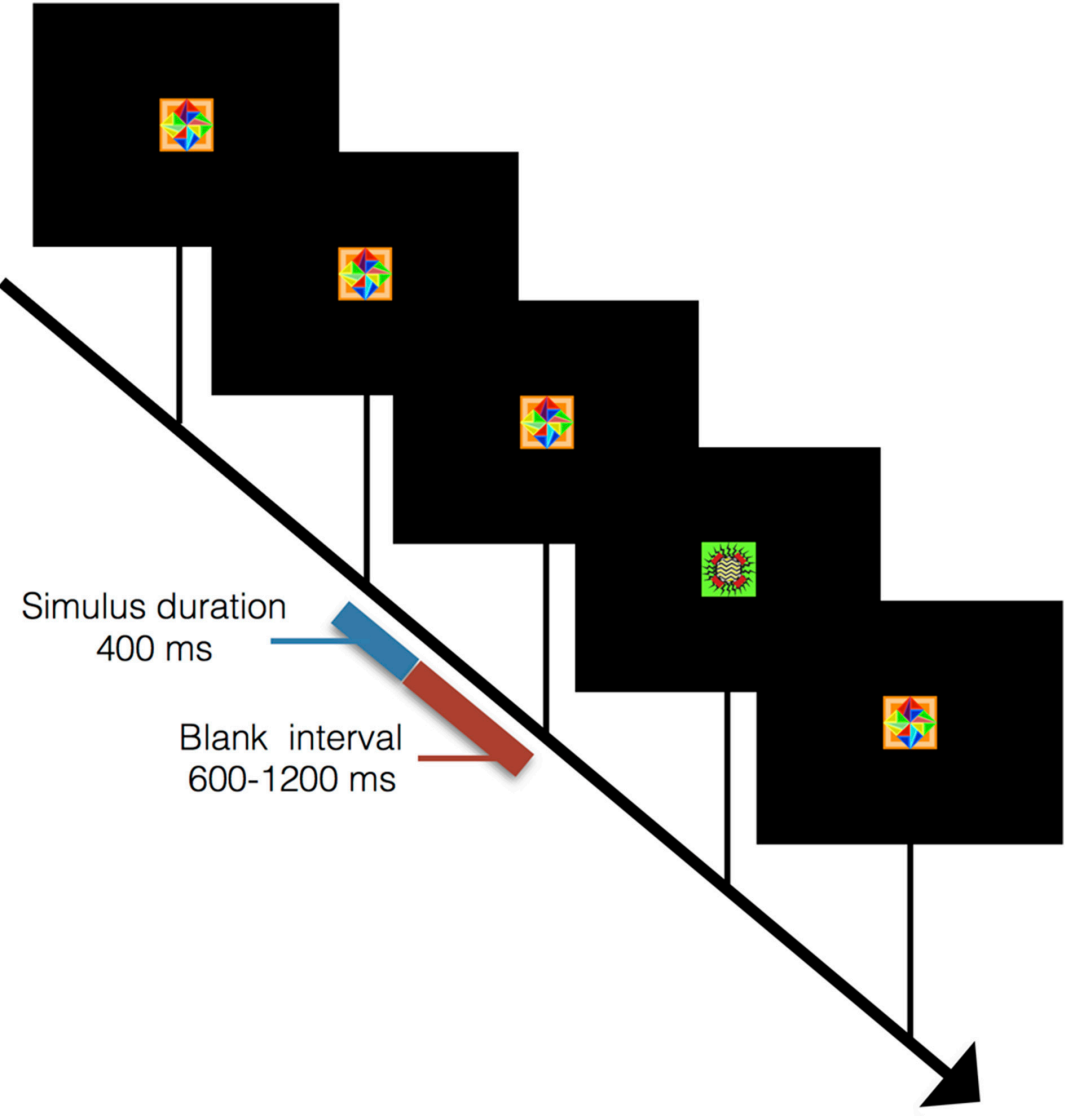
**Outline of visual oddball task**. Sequence for visual oddball task where subjects mentally counted instances of rare stimuli. Stimulus duration was 400 ms with a blank screen lasting between 600 and 1,200 ms between stimuli. A total of 50 rare stimuli were presented interleaved among 200 common stimuli.

In order to isolate the novelty response component, a visual oddball task was also administered where the target stimulus was displayed randomly on 20% of trials. Both targets and distractors were abstract design stimuli presented on a black background screen with an ISI of 1,000–1,600 ms. Stimulus duration was 400 ms (Figure 3). Target stimuli were silently counted while non-target distractor stimuli were to be ignored. For example of prior use of this task see Halgren et al. (1995); further P300 processing details can be found in supplementary materials. Two subjects from the motor task and four other subjects completed this task. This was done to test the novelty response as an explanation for the OFC and posterior parietal responses.

### Data analysis

All electrophysiological and behavioral analyses were conducted offline using custom MATLAB® scripts. Details of line-noise filtering and the multi-taper spectral estimation are all included in Supplementary Materials.

### Condition comparisons

Data for seven anatomical regions were separated into trials with or without a force perturbation in the motor task and with or without a novel visual stimulus in the visual oddball task. For each brain region, differences in the time series data between the task conditions during the 750 ms after movement onset (motor task) and during the 750 ms after the novelty visual stimulus (visual oddball task) were examined by means of a non-parametric cluster statistic (see Statistical Analysis section for more detail). The non-parametric method entailed sampling randomly from trials of all speeds, thus all areas that were found to significantly modulate after perturbations were applied did so regardless of movement speed.

After those areas with significant modulations in response to the perturbations and novelty stimuli were identified, two further analyses were done using the motor task data. The trials where a perturbation was applied were extracted and examined in more detail. The effect of force magnitude on the size of the neural response was examined for each area that showed ERP modulation in the initial analysis. Specifically, perturbation trials were placed in three groups based on the magnitude of perturbation applied: weak (2.5–4.5 N), mild (4.5–10 N), and strong (10–15 N). Differences in these groups were then compared with two-tailed *t*-tests. In addition, the magnitude of the ERP response in the anterior vs. posterior hippocampus was examined relative to matched perturbation magnitudes. This was done to test the hypothesis that the posterior hippocampus is preferentially involved.

### Statistical analysis

#### Perturbation vs. non-perturbation comparison

Significant differences between the time series data in each anatomical region are defined by a non-parametric cluster statistic run on data aggregated from trials by all relevant subjects (Maris and Oostenveld, 2007). This technique has the advantage of implicitly correcting for the multiple comparison problems of many time points. It also leverages the inherent dependency between consecutive time points. The significance of differences between conditions was compared to a null distribution generated by randomly permuting trial condition labels 5,000 times for the motor task. In order to ensure the null distribution had the same anatomical sampling and weighting between sessions as the real data, all permuting of labels was done on a within-session basis. This effectively weights each recording session based on the total number of trials completed in that session. Since parts of the task are self-paced and clinical constraints limit time, the number of trials, rounded to nearest trial, completed in each session varied slightly (142 ± 15). This allowed greater precision in the construction of the null distribution in the face of a comparatively small number of subjects. All simultaneous electrical recordings from within the same anatomical region in a given subject were averaged to help ensure independence of the data points considered.

**Figure 4:**
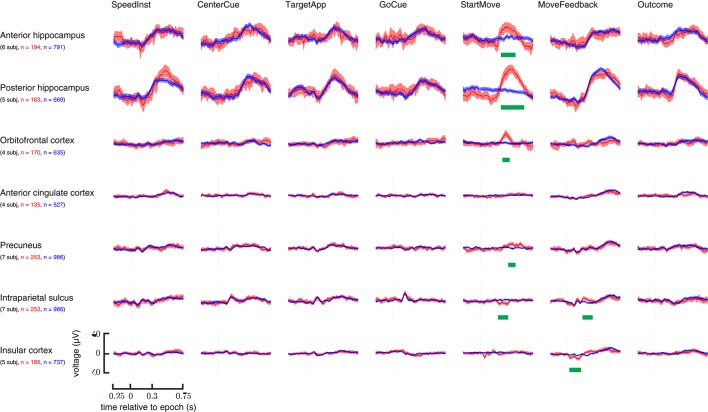
**Overview of all examined brain areas time-locked to each epoch**. Trials with perturbations had them applied at the StartMove epoch. Signals taken from trials with unperturbed movements are in blue; those from perturbed movements are in red. Error bars represent 2 standard errors. N—represents number of total trials contributing to grand average. Statistically significant regions are highlighted with a green bar (See Materials and Methods section for analysis details). Y-axis is in μV.

The standard cluster statistic was employed: the sum of the absolute values of the *t*-statistic stemming from a two-sided *t*-test at each time point after movement onset with an intermediate threshold of *p* ≤ 0.05. Additional details can be found in Maris and Oostenveld (2007). An additional Bonferroni correction was done based on the number of anatomical areas analyzed. Only clusters with uncorrected *p*-values < 0.007 (i.e., *p* < 0.05/7, where seven is the number of anatomical regions) were deemed significant. This was done while looking at the existence of a perturbation induced event (Figure 4).

#### Effect of force on neural correlate size

A series of two-sided *t*-tests were run comparing the ERP responses to different strength stimuli. A bonferroni correction of 15 was done to account for each *t*-test run, resulting in an uncorrected *p*-value threshold of 0.003 (i.e., 0.05/15).

## Results

### Summary of subject details and implanted regions

A total of 12 subjects were involved in our analysis, eight performed the motor perturbation, six performed the visual odd-ball control, and two performed both. Clinical constraints, associated with both fluctuations in subjects' medical conditions and logistical necessities associated with interfacing the experimental apparatus around the bedside allowed more access to some subjects over others. Subject 1 and subject 7 both completed two sessions of the motor task. Table 1 gives further patient details. See Supplementary Table 1 for even more patient-specific performance details. Additional details of tasks are in the Materials and Methods section.

Due to the idiosyncratic nature of the implantation patterns, the brain regions sampled from each subject differed, and thus, the number of subjects contributing to the different analyses varied. Table 2 lists the number of subjects who had contacts in each relevant brain area by task.

### Overview of ERP responsiveness to the different epochs of the motor task

While multiple regions appeared to show some response to early task cues, the two task conditions only meaningfully differed after perturbations' application. Figure 4 lays out these ERPs relative to each epoch in the task.

### ERP responsiveness during perturbed movement

The ERPs of the hippocampus, precuneus, OFC, IPS, insula (IC), and anterior cingulate cortex (ACC) at the onset of movement were examined. Specifically, the difference in neural responses between the perturbed movements and the unperturbed movements were examined. All *p*-values are computed from a non-parametric cluster statistic described in the Materials and Methods section. The anterior hippocampus (286–503 ms, *p* < 0.0002), posterior hippocampus (296–632 ms, *p* < 0.0002), precuneus (404–493 ms, *p* < 0.0002), and orbitofrontal cortex (306–424 ms, *p* = 0.0008) all showed a negative deflection after perturbations were applied. The intraparietal sulcus showed a small positive deflection in the same time range (207–345 ms, *p* = 0.0002). Insula and ACC showed no difference between the conditions (Figure 4; StartMove column).

### Anterior vs. posterior hippocampus ERP response

**Figure 5:**
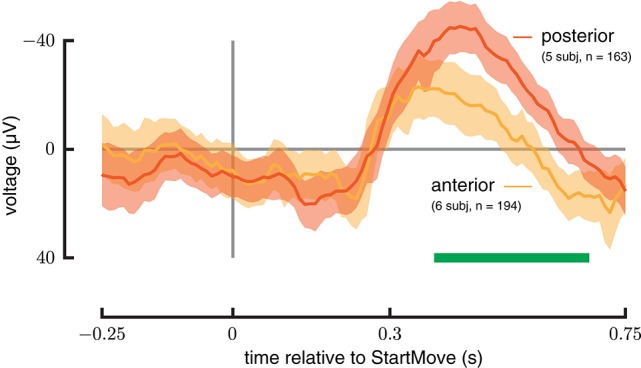
**Anterior vs. posterior Hippocampus**. ERP signals drawn from a force-matched set of trials for anterior (green) and posterior (red) hippocampus during perturbed trials. The posterior hippocampus shows a greater magnitude response. See Materials and Methods section for analysis details.

All recordings taken from the anterior and posterior hippocampus during trials with forces between 8 and 15 N were averaged and plotted in Figure 5 as can be seen, the magnitude of the ERP was significantly larger in the posterior as opposed to anterior hippocampus (385–681 ms, *p* < 0.0002). In addition the peak response latency occured ~100 ms earlier in the anterior as opposed to posterior hippocampus.

### Response to weak and strong perturbations

**Figure 6:**
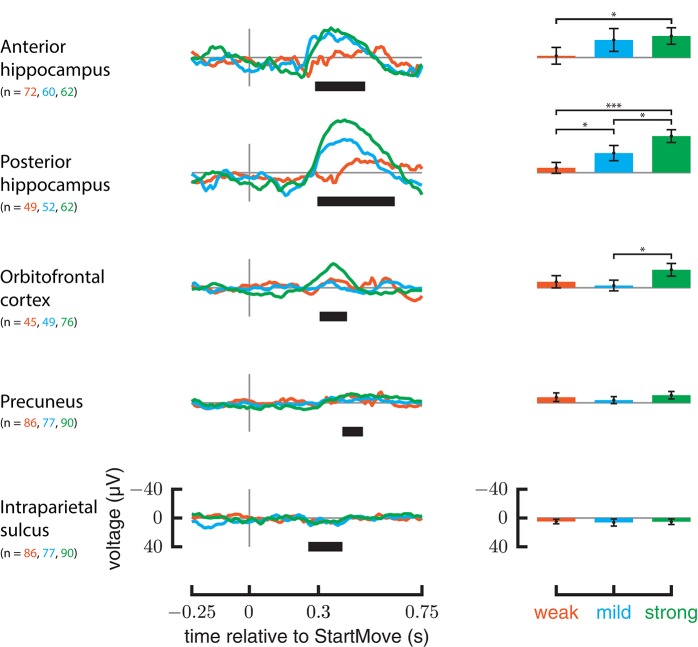
**ERP responses relative to different force magnitudes**. On the left are ERPs for only perturbed trials, grouped by the force magnitude applied. On the rights, the average signal in the significant regions specified in Figure 4 are plotted against force magnitude. Y-axis is in μV. Error markings on bar plot represent 2 standard errors. Stars represent significance of *t*-test after bonferonni correction (1 star— <0.05 level after correction, 3 stars— <0.001 after correction).

Only the anterior hippocampus, posterior hippocampus, and orbitofrontal cortex ERPs show a significant difference between the weakest and strongest perturbations (Figure 6). In the anterior hippocampus the weak forces differed from strong forces (*p* = 0.0011); in the posterior hippocampus all three categories differed from each other (weak-mild *p* = 0.0023, mild-strong *p* < 0.0008, weak-strong *p* < 0.0002). In the orbitofrontal cortex the mild forces differed from strong forces (*p* = 0.0007). Intraparietal sulcus, precuneus, insula, and ACC did not show significant differences between force strengths.

### Comparison with visual oddball control

**Figure 7:**
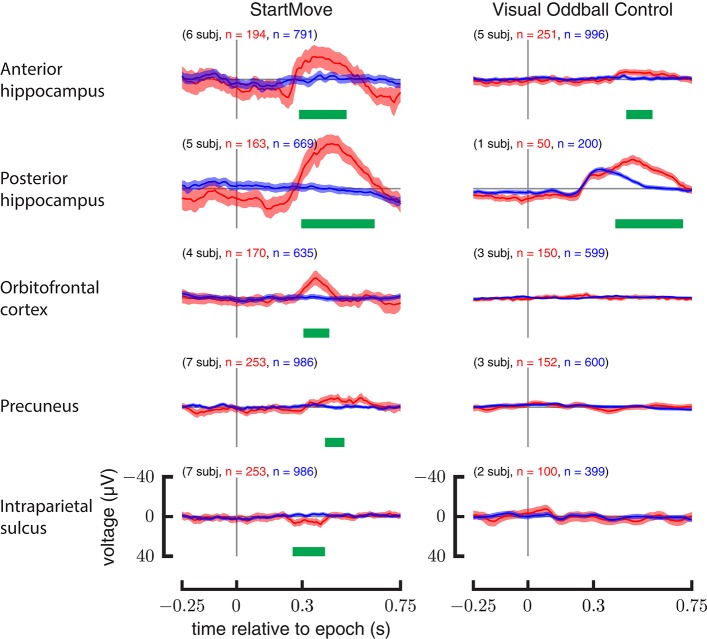
**Comparison of aggregate data for motor task and visual oddball control**. ERPs (left) for perturbed movements (red) and un-perturbed movements (blue). ERPs (right) for the common visual stimuli (blue) and the rare visual stimuli (red) during the visual oddball control task. Y-axis is in μV.

The precuneus, orbitofrontal cortex, and intraparietal sulcus show no significant differences between target (rare) and non-target (common) stimuli. Only the anterior hippocampus (450–570 ms, *p* = 0.004) and posterior hippocampus (400–710 ms, *p* < 0.001) show a statistically significant response. The results are illustrated in Figure 7.

## Discussion

This experiment investigated the role of the associative cortices and hippocampus in goal-directed human motor control when sudden unexpected force perturbations were applied. Working with human subjects in the clinical environment allowed gathering rare high spatial and temporal resolution electrophysiological data. Reaching many brain regions that have seldom been studied during robust motor control makes this a very rare study examining multiple neural effects under these conditions. Responding to large force perturbations during movement can be seen as having three cognitive components, in addition to creating and implementing a motor plan. One, the unexpected nature of the perturbation is detected based on the incoming stimuli's divergence from expectations. Two, among potential responses the correct response is selected while unwanted movements are suppressed. Three, the deviation from expectations drives greater attentiveness to the trajectory of movement.

Prior literature (Wallis et al., 2001; Cavanna and Trimble, 2006; Ludowig et al., 2010) provides indications for the anatomical basis for each of these responses, and we hypothesized the existence of neural correlates of force perturbations in the hippocampus, OFC, and posterior parietal regions. These cognitive responses may supplement the motor planning, error correction, and implementation performed by the pre-motor areas, cerebellum, and primary motor cortex (Scott, 2004). As described below, the analysis of electrophysiological recordings from humans shows patterns consistent with activation of a novelty detection mechanism in the hippocampus, movement plan evaluation in the orbitofrontal cortex, and visuomotor tracking in the posterior parietal regions.

We observed a clear MTL-P300-like response in the anterior and posterior hippocampus in when reaching movements were perturbed. In both rats and humans, the hippocampus has long been known to be involved in both spatial processing and memory (Markus et al., 1994; Eichenbaum, 2004). However, recent work using depth electrodes in humans has shown that the hippocampus responds to visual and auditory oddball stimuli as well. This response, known as the MTL-P300, is believed to be the neural correlate of the novelty response (Ludowig et al., 2010). Other work has shown hippocampus activation during novel somatosensory stimuli (Tarkka et al., 1996). Hence, we hypothesized that the novelty detection mechanism would also be activated during a goal directed movement in response to an unanticipated perturbation.

Additional analysis done by Ludowig et al. (2010) indicated the preferential appearance of the MTL-P300 in the posterior as opposed to anterior hippocampus. This matches the results presented here. Interestingly, while both ERPs began around the same time, the posterior ERP peaked higher and later. The posterior hippocampus also exhibited a comparatively earlier peak on trials with larger perturbations. This may reflect an interesting additional cognitive influence of the manipulation or the attention directed to the recovery from the perturbation. We did not observe this pattern in the OFC response. Both the timing and the valence of the hippocampal data, in addition to the larger magnitude in the posterior portion, strongly support the presence of an MTL-P300 during perturbed reaching movements. The hippocampal activity is most likely related to learning context dependent motor responses with a spatial component as opposed to on-line error correction (Wise and Murray, 1999; Suzuki, 2007). This is supported by human data, where Alzheimer's patients with degraded hippocampi can track a moving object successfully (Eslinger and Damasio, 1986), showing they retain on-line error correction abilities.

The orbitofrontal cortex exhibited an ERP response to force perturbations. Notably, the role of the orbitofrontal cortex in goal directed behavior is still being disentangled. Lesions to the orbitofrontal cortex precipitate reduction in response inhibition (Snowden et al., 2001). In addition, fMRI studies indicate increased activation during tasks that require suppressing behavior (Majid et al., 2013). Tasks negatively impacted by OFC damage include reversal learning (Butter, 1969), devaluation (Pickens et al., 2003), and delay discounting (Mobini et al., 2002). Delayed alternation (Mishkin et al., 1969) and extinction (Bouton, 2004) tasks are also affected. Broadly, the OFC seems crucial for flexible behavior, defined here as subjects changing an established behavioral response to adapt to new contingencies (Schoenbaum et al., 2009). The effect on the detour reaching task is particularly enlightening. In this paradigm, a desirable object is placed within a transparent box with one open facet. When the open facet is toward the participant, a direct reaching movement can obtain the object. However, when the open facet is turned 90° away from the participant, the transparent barrier will deflect a direct reach. The impulse to reach directly must be suppressed and a more circumspect movement plan implemented for success. OFC lesioned primates presented with this task fail, despite repeatedly having their direct reaches blocked (Wallis et al., 2001).

While it is tempting to conclude the OFC is responsible for generating a pure inhibition signal, recent work paints a more nuanced picture. Bryden and Roesch (2015) posit the OFC's involvement in executive function and enhancement of response selectivity when unwanted movements are suppressed and redirected. Schoenbaum et al. (2009) propose the suppression of behavior is really the result of the OFC devaluing the expected reward associated with the initial behavior. The results discussed here do not settle this question: whether the OFC is a source of an inhibitory signal or responsible for reducing the expected reward associated with the initial movement plan. Both ideas, however, point toward the OFC being involved in robust human movement. While recent work has been primarily in rats and non-human primates, we hypothesized the perturbation would necessitate suppressing the prior movement plan and activate executive function in selecting a new motor response. The large OFC ERP shown in this paper strongly supports the involvement of the OFC in responding to unexpected perturbations. Interestingly, both the OFC and hippocampus responses are transient despite the perturbation stimuli remaining constant from beginning to end of movement indicating the responses are not a simple representation of sensory input.

Two parietal areas (intraparietal sulcus and precuneus) both showed ERP responses specific to the force perturbations. These activations in response to the perturbation are interesting, although anticipated in light of existing motor control work. The intraparietal sulcus interfaces between the perceptive and motor systems for controlling arm and eye movements in space (Grefkes and Fink, 2005). The precuneus is involved in directing attention in space (Cavanna and Trimble, 2006). Subjects mentally tracking a subset of bouncing balls while fixating showed increased activation in both the precuneus and IPS during attentive viewing vs. passive observing (Culham et al., 1998). The surprising nature of the perturbation leads to greater attentiveness reflected in increased activity in both cortical areas.

A visual oddball task was administered to determine if the non-hippocampal responses could be explained through only the novelty detection mechanism. Of the four areas shown activating in response to the rare motor perturbations, only the hippocampus showed preferential activation during the visual oddball task. This finding further supports the hypothesis that the hippocampal response is part of a modality-independent novelty response. In addition, the lack of response in the OFC, precuneus, and IPS, despite their innervation with the hippocampus, supports our hypothesis that the separate neural mechanisms described above, are responsible for their activation. Slight differences in the posterior hippocampus MTL-P300 response between tasks may stem from the additional working memory component of the visual oddball task as the subject keeps a running count of rare stimuli.

While each of the cognitive responses discussed here have an existing body of work, they have never been shown to be simultaneously present during perturbations applied to otherwise normal reaching movements. Understanding the neural basis of human's ability to produce rapid, accurate movements even in the face of unexpected perturbations will require elucidating the synergies between the associative cortices and the primary motor system.

While it is worth noting that the subjects of this study are not completely healthy individuals, there are good reasons to believe they can be proxies for understanding the ordinary healthy brain in this context. Subjects like this have been the basis of multiple electrophysiological studies in other contexts (Ossandón et al., 2011; Morillon et al., 2012; Yaffe, 2015). These signals are present outside the resected regions, and the patient's individual symptoms indicate the primary motor system is not involved. Subjects are able to complete the movements in the allotted time on almost every trial and exhibit qualitatively normal movements, indicating adequate neural circuitry is in place. As an additional note, if it were discovered that neural correlates described here were modified by the location of the epileptic focus, that information could ultimately prove invaluable in guiding the very brain resection procedure that brought the subjects into the hospital environment.

With this said, we also note the limitations of this study. We had relatively few patients performing each task, and even fewer performing both the visual and motor tasks. This is because not all patients consented and/or met the criteria of our study; and, some patients were uncomfortable with some aspects of the motor task. In particular, different patients had different tolerance levels for force perturbation amplitudes. This did not allow us to investigate responses to force perturbations that ranged over the same amplitudes across all patients. The small sample size of the study population is further limited by the fact that each patient had electrodes implanted in different brain regions.

## Author contributions

MK, SS, JTG, and KK were involved in project development. MK, JTG, MJ, HP, and KK were involved in experimental setup. JGM, JB, and JTG were involved with patient recruitment. JGM, JB, JL, JJ, MJ, HP, ST, JTG, MK, KK, and CL were involved with data collection. ST, MJ, PS, and HP were involved with data preprocessing. PS, JJ, JL, MK, KK, SS, CL, and JTG were involved with data analysis and interpretation. All authors were involved in manuscript preparation.

## Funding

This work was supported by a National Science Foundation grant (EFRI-MC3: # 1137237) awarded to SS, JGM, JB, and JTG. Also supported by the ARCS Foundation, the Kavli Foundation, and the NSF GRFP (DGE-1232825).

### Conflict of interest statement

The authors declare that the research was conducted in the absence of any commercial or financial relationships that could be construed as a potential conflict of interest.
