# Supplementary material for: The Role of Associative Cortices and Hippocampus during Movement Perturbations
Source: Front Neural Circuits. 2017 Apr 19;11:26. doi: 10.3389/fncir.2017.00026 (PMC5395558; doi:10.3389/fncir.2017.00026)
Supplement: Supplementary file 1 [file DataSheet1.pdf]

frontiers

Supplementary Material

The Role of Associative Cortices and Hippocampus during Movement Perturbations

Matthew Kerr PhD[1], Pierre Sacre PhD[1], Kevin Kahn PhD[1], Hyun-Joo Park PhD [2], Mathew Johnson BS [3], James Lee BS [3], Susan Thompson MS [3], Juan Bulacio MD [4], Jaes Jones BS[3], Jorge Gonzalez-Martinez MD, PhD[4], Catherine Liegeois-Chauvel[4,5,6], Sridevi V. Sarma PhD [1,†], John T. Gale PhD [2,3,†,*]

[1] Department of Biomedical Engineering, Johns Hopkins University, Baltimore, MD, USA 21218

[2]Center for Neurological Restoration, Cleveland Clinic, Cleveland, OH, USA 44195

[3] Department of Neuroscience, Cleveland Clinic, Cleveland, OH, USA 44195

[4] Epilepsy Center, Cleveland Clinic, Cleveland, OH, USA 44195

[5] INSERM UMR 1106, INS 1106

[6]Aix Marseille University, Marseille, France

[†] These authors contributed equally

*** Correspondence:** Corresponding Author: msdk2@cam.ac.uk

1 Supplementary Methods

Data Analysis and Filtering Details

For ERP analysis, a second-order IIR notch filter was applied to the raw SEEG data at 60 Hz and its harmonics: bandwidth of 3Hz at -1dB suppression. A high-pass 5th order Butterworth filter was also applied to detrend the data with a cutoff of 0.5 Hz, followed by decimating the data to 100 Hz. The 750 ms after each event was extracted, time-locked, and averaged across trials to examine the P300 effect.

On a macro level contacts on the clinical electrodes that were obviously faulty or showed large artifacts were disregarded entirely. Subsequently, all recorded epochs were included in this analysis. No data was rejected on a trial-by-trial basis.

For high frequency activity analysis, the same filtering was applied with the exception of the decimation. Multi-taper spectral estimation[1] was then performed with a time window of 300 ms, a time step of 5 ms, three tapers, and a time-bandwidth product of 2. The high frequency activity (HFA) metric captures high gamma activity and reflects previous work in SEEG[2] and other invasive recordings. It puts equal weight on all frequencies between 50 and 150 Hz and corrects for the 1/f falloff in power. It is calculated by first taking the natural log of the power in each frequency, then

performing a Gaussian normalization based on the power in each frequency over the entire recording session. In order to limit the effect of outliers, only data from the 5th to 95th percentile were used when calculating the mean and variance for the normalization procedure. HFA includes the average of all normalized log-power frequency bins between 50 and 150 Hz at each time point. Faulty recording contacts, or contacts with interictal activity, were identified by visual inspection and disregarded.

Calibration Details

A series of five reaching movements towards a target to the right of the center position was completed while the subject was instructed to reach as quickly as possible. The shortest time to target in these trials was used to derive the subject-specific maximum movement speed. The fast and slow movement time instructions provided in the main task were based on 1/3 and 2/3 of the subject specific maximum speed to target, placing a limit on the time the subject can take to reach the desired target and still receive a reward cue. This was done to encourage a range of movement speeds across trials.

2 Supplementary Results

HFA Responsiveness during perturbed movement

HFA responses after the start of movement show similar albeit not identical results. Posterior Hippocampus (151 – 701 ms, $p < .0002$), Precuneus (251 – 701 ms, $p = 6e-4$), and IPS (301 – 701 ms, $p = 0.0038$) all show an HFA response. The OFC trend is there, but falls with a p-value of 0.011 falls just short of Bonferoni corrected significance. IC (101 – 701 ms, $p < 0.0002$) shows an HFA response that is not captured in the ERP. See supplementary materials for plots.

3 Supplementary Figures and Tables

Below is a figure representing the high frequency activity after the start of movement.

3.1 Supplementary Figures

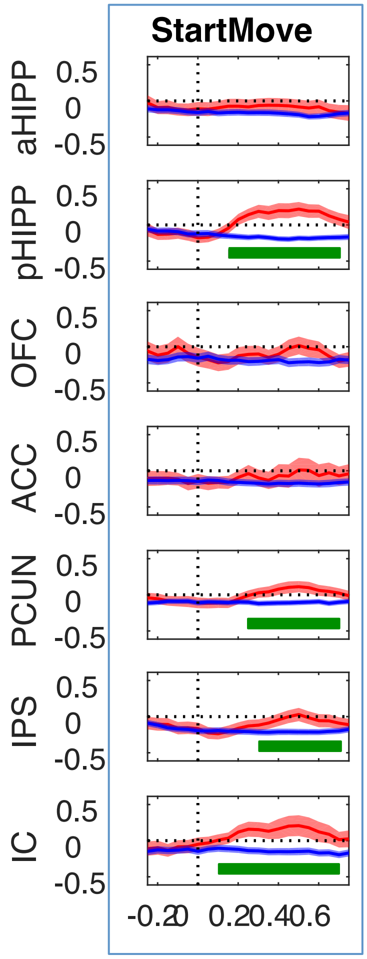

Supplementary Figure 1. HFA analysis of each brain regions aligned to all cues. Rectangular bars represent times of significant difference as identified as non-parametric cluster statistic. X-axis is time from cue. Y-axis is voltage in uV.

3.2 Supplementary Tables

Supplementary Table 1: Columns 2-4 represent only completed trails

Patient	Trials with perturbation	Trials without perturbation	Aggregate Success Rate	Completion Rate
Subject 1	27<br><br>26	105<br><br>98	56%<br><br>53%	98%<br><br>100%
Subject 2	30	113	66%	92%

Subject 3	26	128	30%	94%
Subject 4	24	115	50%	97%
Subject 5	31	122	53%	96%
Subject 6	29	123	70%	99%
Subject 7	25	102	29%	93%
Subject 8	30<br>30	94<br>88	59%<br>64%	96%<br>98%
Subject 9	N/A	N/A	N/A	N/A
Subject 10	N/A	N/A	N/A	N/A
Subject 11	N/A	N/A	N/A	N/A
Subject 12	N/A	N/A	N/A	N/A

4 References

1. Mitra, P. & Bokil, H. *Observed Brain Dynamics*, Oxford University Press, New York (2007)
2. Ossandón, T., Jerbi, K., Vidal, J. R., Bayle, D. J., Henaff, M. A., Jung, J., Minotti, L., Bertrand, O., Kahane, P. & Lachaux, J. P. Transient suppression of broadband gamma power in the default-mode network is correlated with task complexity and subject performance. *J. Neurosci.* **31**, 14521–14530 (2011)
